# Informing Content and Feature Design of a Parent-Focused Human Papillomavirus Vaccination Digital Behavior Change Intervention: Synchronous Text-Based Focus Group Study

**DOI:** 10.2196/28846

**Published:** 2021-11-19

**Authors:** Elisabeth RB Becker, Ross Shegog, Lara S Savas, Erica L Frost, C Mary Healy, Stanley W Spinner, Sally W Vernon

**Affiliations:** 1 The University of Texas Health Science Center at Houston Houston, TX United States; 2 Baylor College of Medicine Houston, TX United States; 3 Texas Children's Pediatrics Houston, TX United States

**Keywords:** human papillomavirus, vaccination, qualitative, focus groups, sexually transmitted infection

## Abstract

**Background:**

Human papillomavirus (HPV) is a common and preventable sexually transmitted infection; however, vaccination rates in the United States among the target age group, which is 11-12 years, are lower than national goals. Interventions that address the barriers to and facilitators of vaccination are important for improving HPV vaccination rates. Web-based, text-based focus groups are becoming a promising method that may be well suited for conducting formative research to inform the design of digital behavior change intervention (DBCI) content and features that address HPV vaccination decision-making.

**Objective:**

This study aims to explore parental HPV vaccination decision-making processes using a web-based, text-based focus group protocol to inform content and feature recommendations for an HPV prevention DBCI.

**Methods:**

We conducted 4 web-based, text-based synchronous focus groups via Skype with the parents of patients aged 11-13 years within a large urban US pediatric clinic network.

**Results:**

The 22 parents were mostly female, White, non-Hispanic college graduates, and they mostly had private health insurance for their children. Approximately half (14/25, 56%) of the parents' 11-13 year old children had initiated HPV vaccination. Most parents had experience using Skype (19/22, 86%). Approximately half (8/17, 47%) of parents expressed no preference for the focus group format, whereas 47% (8/17) requested a text-only chat format and 6% (1/17) requested an audiovisual format. The three main themes from the qualitative data were barriers to HPV vaccination, facilitators of HPV vaccination, and suggestions for improving the HPV vaccination clinic experience. A total of 11 intervention content and feature recommendations emerged from the themes, including addressing HPV knowledge barriers using trusted sources, designing for a family audience, focusing on the framing of messages, reporting reputable HPV research in a comprehensible format, and expanding the clinic visit experience.

**Conclusions:**

Synchronous text-based focus groups are feasible for conducting formative research on HPV vaccination decision-making. Among well-educated and well-resourced parents, there are barriers such as misinformation and facilitators such as pediatrician recommendations that influence HPV vaccination decision-making. Parents want to conduct their own HPV research as well as receive relevant HPV vaccination advice from their child’s pediatrician. In addition, parents want an enhanced clinic visit experience that lets them access and connect to tailored information before and after clinic visits. The results gathered provide guidance for content and features that may inform a more responsive DBCI to address HPV vaccination decision-making among parents.

## Introduction

### Background

Human papillomavirus (HPV) is the most common sexually transmitted infection (STI) in the United States [1] and worldwide [2]. The US Centers for Disease Control and Prevention (CDC) estimates that approximately 90% of men and 80% of women will be infected with at least one type of HPV in their lives [3]. HPV infection generally occurs within a few months to years of becoming sexually active [4,5].

HPV can cause asymptomatic infections, warts, and cancer in women and men [6]. An estimated 34,800 HPV-attributed cancers of the cervix, vagina, vulva, penis, anus, and oropharynx are diagnosed in the United States every year [7]. Most of these cancers are associated with the infection of HPV types 16 and 18. Although HPV is commonly associated with cervical cancer, its prevalence in oropharyngeal tumors has increased substantially from the 1980s (16%) to the 2000s (73%) [8]. In addition to cancer, HPV causes 300,000 new cases of genital warts each year via HPV types 6 and 11 in the United States [9]. Owing to HPV’s prevalence and ease of transmission, there have been global efforts to prevent the spread of HPV infection.

Historically, 3 prophylactic HPV vaccines (2vHPV, 4vHPV, and 9vHPV) have been licensed for use in the United States since the first HPV vaccine became available in 2006 [10,11]. Over time, the dosing regimen, range of protection, and intended patient profile for the HPV vaccine have changed. Since 2016, the 9-valent HPV vaccine (9vHPV) has been the only HPV vaccine sold in the United States [12] and is currently available for males and females aged 9-45 years. The Advisory Committee on Immunization Practices recommends that routine HPV vaccination be initiated for children aged 11-12 years [13]. The Healthy People 2030 goal for HPV vaccination series completion is 80% of adolescents aged 13-15 years. However, national samples of adolescents aged 13-17 years estimate that only 60% have initiated and 40% have completed the HPV vaccination series [14].

The modification of factors that negatively affect parental vaccination decision-making is a key strategy to improving HPV vaccination rates [15,16]. A 2019 systematic review of 41 US-based studies exploring HPV vaccine beliefs found four negative beliefs—perceived adverse effects (ie, promotes sexual activity, too new, and causes illness), perceived lack of necessity (not sexually active), morality concerns (stigmatizing recipients), and skepticism about effectiveness—and one positive belief, that it prevents STIs, across the literature [17]. In comparison, the National Immunization Survey-Teen 2010-2016 trend data found safety concerns, a lack of vaccine knowledge, and perceived lack of necessity as consistent reasons why parents did not initiate HPV vaccination for their adolescents [18]. Among caregivers, the internet is a popular source for information regarding HPV vaccination [19]; however, most websites that contain information about HPV immunization have poor readability [20]. Furthermore, web-based consumer health information is susceptible to miscommunication, misrepresentation, and misappropriation, which can result in negative health consequences for information seekers and those they care for [21]. HPV prevention digital behavior change interventions (DBCIs) that focus on relatable, understandable, and actionable information may facilitate HPV vaccination decision-making among parents.

Formative research constitutes an important component informing DBCI content and feature design [22-25] and includes understanding the barriers and facilitators of vaccination in the context of HPV. As the adaptation of research protocols to remote formats becomes imperative [26,27], web-based, text-based focus groups may serve as a feasible way to gather DBCI formative data. Web-based synchronous text-based focus groups have addressed a range of issues, including parental attitudes toward the 9-valent HPV vaccine [28], HPV mobile health preferences among young men who have sex with men [29], and views on sex among childhood survivors of cancer [30,31]. Studies comparing web-based synchronous text-based focus groups with the traditional in-person approach have found similar thematic content and quality across the formats [32-34].

### Objective

The purpose of this study is to explore parental HPV vaccination decision-making processes within a large urban pediatric clinic network using a web-based, text-based focus group protocol to inform content and feature recommendations for an HPV prevention DBCI. Specifically, we seek to answer the following three research questions:

1. Themes: What are the influences of HPV vaccination decision-making among parents belonging to a large pediatric clinic network?
2. Intervention recommendations: On the basis of the themes, what are the content and feature recommendations for a DBCI to encourage HPV vaccination?
3. Format feasibility: How feasible is conducting formative HPV research with parents using synchronous text-based focus groups?

## Methods

### Overview

Four 60-minute web-based synchronous text-based focus groups were conducted via Skype (Microsoft Inc) in October 2016 with parents of adolescents aged 11-13 years. The adolescents were patients at a large urban and geographically diverse pediatric clinic network in Texas, United States. Parents were invited to participate in the focus groups through their pediatricians, study recruitment flyers posted in the clinic waiting rooms, and advertisements on the clinic’s Facebook page. Recruited parents completed a phone-based screening to assess their eligibility. After verbal consent was received, potential parents were sent a demographic and focus group preference survey. Parents were provided a Skype username and password for the duration of the study along with a Skype guide that included download and log-in instructions with screenshots. Usernames were created that included the parent’s first name and an ID number and were deleted at the conclusion of each session. Parents were instructed to use Skype on a laptop or desktop, so their mobile phone would be available if tech support was needed. Approximately 48 hours before the start of their session, parents were asked to log into their study account and answer the question, “What activities do you like to do with your child or children on the weekend?” as a check that they had successfully downloaded Skype (if needed) and could navigate the chat function. A total of 4 research staff members conducted the synchronous focus groups. This included the lead moderator who posted questions in the group chat, 2 submoderators who took notes and monitored the discussion for opportunities to probe participants, and a technology facilitator who assisted parents with using Skype if needed. A US $40 web-based gift card incentive was offered to the parents at the completion of their focus group. Study protocols and procedures were approved by the University of Texas Health Science Center at Houston institutional review board (HSC-SPH-15-0202).

### Participant Inclusion Criteria

Participants were eligible for the study if they were a parent or legal guardian of a patient aged 11-13 years in the clinic network. Participants needed an internet connection, access to a desktop or laptop with a keyboard, and the ability to download and use the free web-based video conferencing and chat platform, Skype [35]. Participants also needed to be able to read and write in English.

### Measures

#### Demographics

Parents’ demographic variables included sex, age, race, ethnicity, education, number of children, child’s health insurance status, child’s HPV vaccination initiation status, and adolescent vaccine hesitancy status. Parent-adolescent vaccine hesitancy was assessed using an adapted question from the Parent Attitudes about Childhood Vaccines survey [36], “Overall, how hesitant about adolescent vaccinations (HPV, Tdap [tetanus, diphtheria, and pertussis], Meningitis, and influenza), would you consider yourself to be?” with Likert-scale response options of *not hesitant*, *somewhat hesitant*, *unsure*, *hesitant*, and *very hesitant*.

#### Skype Use

Skype use was assessed by asking parents how often they use Skype (aware but never use, use sometimes, or use regularly). The preferred Skype focus group format was assessed by asking parents how they would like to communicate for the focus group (Skype chat [text-based only], Skype call [audio and visual], or no preference). Skype logistics were evaluated from the no-show rate for each focus group session and the difficulty in attending the session (ability to log into the account 48 hours before the session, being late to the session, calls with the technology facilitator, and technical difficulties during the session). The costs of using the web-based Skype format were compared against the projected costs of conducting in-person focus groups at the clinics. Although both groups included participation incentive costs, in-person groups would have required additional costs, including transcription, parking reimbursements, and after-hours pay of clinic staff.

### HPV Vaccine Decision-Making

Research staff trained in qualitative research conducted the focus groups using a discussion guide comprising questions on parental vaccination attitudes and vaccination decision-making processes (Textbox 1). Topic 3 included a series of DBCI subquestions. The discussion guide was informed by prior qualitative research conducted with the network clinic staff and HPV vaccination barriers and facilitators reported in prior research [28,37-43]. Optional probes were generated beforehand and modified in real time as needed.

**Focus group guide.**

**Topic 1**

- Prompt: Patients aged 11-12 years typically receive four vaccinations: Tdap, meningococcal, human papillomavirus (HPV), and flu. In the survey you completed, some of you indicated that your adolescent has received all of these vaccinations, whereas others indicated that your adolescent has not received one or more of these vaccinations.
- Question: How do you decide if your adolescent should receive certain vaccinations? Can you please describe that process?

**Topic 2**

- Prompt: At the network clinics, rates of HPV vaccination are lower than rates of the other adolescent vaccinations. In the survey you completed, some of you said that your adolescent has received the HPV vaccine and some of you said that your adolescent has not yet received the HPV vaccine.
- Question: What were the most important factors in making the decision whether to vaccinate or not vaccinate your adolescent for HPV?

**Topic 3**

- Prompt: In light of what we have just discussed, let us consider ways to improve the parent experience at the clinic.
- Question: How can the clinic help you as a parent in making decisions around vaccinating your adolescent against HPV?
- Digital behavior change intervention subquestions:
  How can the clinic best communicate with you about HPV? What channels?
  Would you like to receive information from the clinic through a phone app? Why or why not?
  What educational information about the HPV vaccine on a website or phone app would be most helpful to you?

### Data Analysis

Demographics were analyzed descriptively, including mean and range for continuous data and frequency and percentage for nominal data. Skype logistics data were analyzed from focus group transcripts and call logs. Cost savings were calculated by comparing the estimated in-person costs of focus groups with the costs of Skype-mediated focus groups. The qualitative analysis was completed in two phases. The first phase involved 4 project staff members creating a preliminary codebook and assessing the frequency of responses to specific questions across the focus groups. The second phase involved the lead moderator (first author, ERBB) conducting a conventional content analysis, the systematic classification process of coding and identifying themes, with coding categories derived directly from the data [44]. In the review of each transcript and accompanying notes, existing codes and broader categories were used to identify themes for parental vaccination decision-making. These findings further informed the content and feature design recommendations of an HPV prevention DBCI [45]. The identified themes were reviewed and retained on the basis of the consensus of the research team.

## Results

### Demographic Characteristics

A total of 22 parents with an adolescent aged 11-13 years participated in the web-based, text-based focus groups (Table 1). Parents came from 31% (16/51) of clinics within the network. There were 4-7 parents in each session. Parents were aged 41.9 years (SD 6.1 years); had an average of 2 children; were mostly female (21/22, 95%), White, and non-Hispanic (13/22, 59%); had a graduate or professional degree (10/22, 45%) and had private health insurance for their child or children (18/22, 82%). Most parents were not hesitant (11/22, 50%) or somewhat hesitant (8/22, 36%) toward adolescent vaccinations. Approximately 56% (14/25) of the parents' 11-13 year old children had initiated HPV vaccination. This approximates the demographic characteristics of the clinic network population, where, among children aged 10-17 years, 45% (56,934/127,975) were White and non-Hispanic, 80% (102,223/127,975) had private health insurance, and 58% (74,204/127,975) had initiated the HPV vaccination.

**Table 1:** Parent demographics (N=22).

Characteristic			Study participants
**Parent age (years)**			
	Value, mean (SD)		41.95 (6.12)
	Value, range		30-52
**Number of children^a^**			
	Value, mean (SD)		1.95 (1.31)
	Value, median (range)		2 (1-5)
	Value, mode		1
	Total male		19
	Total female		24
**Parent sex, n (%)**			
	Male		1 (5)
	Female		21 (95)
**Parent race and ethnicity, n (%)**			
	White and non-Hispanic		13 (59)
	Black or African American and non-Hispanic		7 (32)
	Hispanic^b^		2 (9)
**Parent education, n (%)**			
	High school graduate		4 (18)
	College graduate		8 (36)
	Graduate or professional degree		10 (45)
**Child or children’s health insurance status, n (%)**			
	Private health insurance		18 (82)
	Medicaid		2 (9)
	State Children’s Insurance Program		1 (5)
	Military health care		1 (5)
**Adolescent vaccination hesitancy status^c^, n (%)**			
	Very hesitant		2 (9)
	Hesitant		1 (5)
	Unsure		0 (0)
	Somewhat hesitant		8 (36)
	Not hesitant		11 (50)
**Child or children’s HPV^d^ vaccination initiation status^e^, n (%)**			
	**9-10 years**		
		Yes	0 (0)
		No	5 (100)
	**11-13 years**		
		Yes	14 (56)
		No	11 (44)
	≥**14 years**		
		Yes	10 (77)
		No	3 (23)
**Skype use, n (%)**			
	Aware of Skype but never used it		3 (14)
	Use sometimes		18 (82)
	Use regularly		1 (5)
**Preferred focus group format^f^, n (%)**			
	Skype chat (text-based only)		8 (47)
	Skype call (audio and visual)		1 (6)
	No preference		8 (47)

^a^Children aged 9-23 years.

^b^A total of 2 parents only reported Hispanic ethnicity and no racial category.

^c^Includes human papillomavirus, Tdap (tetanus, diphtheria, and pertussis), meningitis, and influenza vaccination.

^d^HPV: human papillomavirus.

^e^n=43 children; received at least one human papillomavirus vaccine dose.

^f^Data missing for 5 parents.

### Influences of HPV Vaccination Decision-Making: Qualitative Findings

Three themes emerged regarding parents’ HPV vaccination decision-making processes: (1) barriers to HPV vaccination, (2) facilitators of HPV vaccination, and (3) suggestions for improving the HPV vaccination clinic experience.

#### Barriers to HPV Vaccination

Barriers that affected parents’ HPV vaccination decision-making were driven by HPV misinformation and confusion, negative HPV beliefs and attitudes, and navigating trustworthy HPV information on the internet. Parents were unsure if boys needed or were eligible for the HPV vaccine. There was also concern that the vaccine did not cover all the “mutations of the virus” and that the “virus changed structure over time”:

The HPV vaccine does not cover enough (too many types) for me to inject my son with some unknown drug. I feel like they are trying to scare us into doing it. I am not sold. [Female, 45 years, Black, non-Hispanic, very vaccine hesitant] (1)

A parent who had chosen not to vaccinate her son for HPV expressed the hope that people would get tested for STIs (including HPV) before having sex, although there is no Food and Drug Administration–approved HPV test for males. Another parent thought HPV was linked to herpes (although HPV does not cause herpes, it can cause genital warts). Parents questioned why “the vaccine series is not effective once you pass a certain age” and “does it work for those who are older than 26 but not sexually active?”

Parents reported negative beliefs and attitudes toward the HPV vaccine. Many parents who considered themselves generally provaccination were skeptical about the HPV vaccine as they perceived it to be new and possibly have long-term negative health effects:

We are not getting it [HPV vaccine] right now. Maybe in a few years, when my daughter is closer to becoming sexually active and that way, after a few more years have gone by, the vaccine will be more tested and better. [Female, 43 years, White, non-Hispanic, not vaccine hesitant] (2)

The belief of being able to postpone the HPV vaccine because of the perceived sexual inactivity of their adolescent was echoed by other parents:

I am reluctant to start the HPV shots with my soon to be 13-year old son because I don’t think it’s necessary at this moment to start it with him and I need to do more research to learn more about the risks of doing it or not doing it. [Female, 40 years, White, non-Hispanic, somewhat vaccine hesitant] (3)

I have always given my daughter the expected and recommended vaccinations except the HPV vaccination because of concerns I heard about negative effects, and also knowing absolutely that she is not sexually active yet I felt I had time to do some research and make an informed decision. [Female, 50 years, White, non-Hispanic, somewhat vaccine hesitant] (4)

Parents also questioned if the vaccine was being promoted for financial gain:

I just wonder if the doctors are urging these vaccines because it is what they believe is best or is big pharm pushing it. [Female, 45 years, Black, non-Hispanic, very vaccine hesitant] (5)

Parents generally felt the need to do their own HPV research, even when provided with recommendations by health care providers:

My pediatrician brought it [HPV vaccination] up at her last year’s checkup (when she turned 12). She gave me a handout about the [HPV] vaccine and accepted my response that I was not ready to commit to it yet but wanted to research more and think about it. She did say she recommended it, but did not push, at least not at that visit. [Female, 50 years, White, non-Hispanic, somewhat vaccine hesitant] (6)

When doing their own HPV research, trustworthy information sources differed among parents. Although pharmaceutical advertisements and beliefs of the general public were seen as untrustworthy sources of HPV information and pediatricians were overwhelmingly seen as trustworthy HPV sources, there was little agreement among other sources. For example, family and friends, the internet, and news articles were HPV information sources that parents had differing opinions about:

TV news, old fashioned newspapers, and NPR are my primary sources for information. Generally they touch on a vaccine when it is new, recalled, or if there is a bump in a disease spreading. Not social media, not friends, not internet. I do read all the fine print in printed RX ads. [Female, 47 years, White, non-Hispanic, not vaccine hesitant] (7)

CDC website, friends and family, other news articles from magazines or website that are informative. I don’t take my info from add in tv or magazine since they are paid by the pharmaceutical industry.... [Female, 45 years, White, non-Hispanic, somewhat vaccine hesitant] (8)

Some parents noted the CDC and National Institutes of Health as trustworthy HPV information sources, whereas others commented that they had never thought of using these agencies:

...for some reason, I never thought to check the cdc about hpv, but after this focus group that makes a lot of sense...thank you all who have mentioned that [Female, 37 years, Black, non-Hispanic, vaccine hesitant] (9)

A parent also expressed knowing a CDC scientist who was not in support of the HPV vaccine. Navigating *reputable research* on the internet proved particularly challenging for many of the parents. This was highlighted in an exchange during a focus group where one parent shared a link to an article written by a physician disputing HPV vaccine effectiveness that *freaked her out,* and another parent provided a website citing that physician and his organization as fraudulent and not scientifically sound [46].

Overwhelmingly, parents had misinformation about HPV and the HPV vaccine and had differing opinions about *go-to* sources for reputable information. The HPV vaccine was seen as different from other adolescent vaccinations, even among those that considered themselves provaccine, because of the perceived newness, perceived lack of evidence, and belief that the vaccine could be postponed until a child is sexually active.

#### Facilitators of HPV Vaccination

Facilitators of HPV vaccination included positive HPV beliefs and attitudes, family members and close friends experiencing negative health outcomes from preventable illness, personal experience with HPV, and pediatrician recommendations. Positive beliefs about the HPV vaccine centered around decreasing their children’s risk of acquiring STIs and cancer, protecting their children’s future partners, and protecting public health. These beliefs were highlighted when parents were asked what comes to mind when they hear *HPV vaccine,* and statements around *cancer and sexually transmitted disease prevention* were the most prominent. Parents expressed that the HPV vaccine provided a unique opportunity to prevent cancer:

*Thank God for Modern Medicine. With technology and medical advancements, why are we not excited about the ability to PREVENT cancer- not just CURE it? Let’s educate our parents and help them advocate for their children- it’s time to be proactive now, rather than reactive later.* [Female, 43 years, White, non-Hispanic, not vaccine hesitant] (10)

Most parents expressed that their child could be at risk of contracting HPV in the future. Parental perceived risk often centered on future partners:

As I said before, my hope is that my children choose to abstain from sex until marriage. Should they choose not to do that, I hope they use safe sex practices. But even if they did all the “right things,” they still might wind up married to someone who was exposed to HPV. [Female, 37 years, White, non-Hispanic, not vaccine hesitant] (11)

Absolutely. Unfortunately, I think STD are very widespread. And I don’t think people are generally forthcoming with telling others that they have an STD before having sex. [Female, 40 years, White, non-Hispanic, somewhat vaccine hesitant] (12)

Concerns over asymptomatic HPV infection and rape were also included in the discussion about risk. Parents mentioned that unlike other vaccines, HPV made them confront the impending adulthood of their adolescents:

...this is a vaccine that comes with a recognition that your child will someday be an adult. [Female, 44 years, White, non-Hispanic, somewhat vaccine hesitant] (13)

There is far more negative press associated with HPV and associating it with sexually transmitted disease. No one wants to think of their prepubescent child that way. It needs to be seen as a positive advancement in pediatric medicine. [Female, 43 years, White, non-Hispanic, not vaccine hesitant] (14)

Having a family member or close friend experience a negative health outcome related to HPV or another vaccine-preventable illness significantly influenced the way parents felt about the HPV vaccine:

I did not want to vaccinate my sons for HPV (even though it might be the best thing for public health) until I learned of a brother of a good friend who is gravely ill from HPV related throat cancer. [Female, 52 years, White, non-Hispanic, somewhat vaccine hesitant] (15)

One parent had HPV-attributed oropharyngeal cancer and became a vocal advocate for the HPV vaccine:

I know my initial rejection of the vaccination for my oldest son was that I was not raising him to be promiscuous, so I figured there was very little chance of him being exposed. Once I read the incredibly high transmission rates for the HPV virus and how easily it is spread, and got to experience firsthand what the treatment was like, my oldest son was at the doctor receiving the vaccine immediately. I personally try to educate every parent I meet with younger children if the conversation gets steered to that subject. Usually all it takes is showing them my 5 inch scar from my neck dissection and relating how my taste buds are permanently destroyed from the radiation. [Male, 45 years, White, non-Hispanic, not vaccine hesitant] (16)

Overwhelmingly, parents expressed that a recommendation from their child’s pediatrician positively influenced their decision to give their child the HPV vaccine, particularly if they were able to do their own HPV research before the clinic visit:

I was not sure about HPV for boys (and due to the fact that it’s pretty new) but decided to follow recommendations from pediatrician and also from several articles that I read. [Female, 45 years, White, non-Hispanic, somewhat vaccine hesitant] (17)

I just recently became aware of HPV for boys from tv commercials and looked into it before my son was of age to receive it. At his last annual check-up, our pediatrician recommended the vaccine and I trusted her judgement enough to agree. [Female, 39 years, Black, non-Hispanic, not vaccine hesitant] (18)

There were additional factors that acted as both a barrier and facilitator to HPV vaccination. For example, adolescents influenced their parents’ HPV vaccination decision-making process and were sometimes given autonomy over the decision:

My son heard a commercial about HPV and he wants to get the vaccine. My mom recently passed away from cancer and so he has a serious concern. [Female, 37 years, Black, non-Hispanic, vaccine hesitant] (19)

My second daughter chose not to receive the vaccine after discussing with our pediatrician. She is extremely mature and I was not going to insist she do something she felt strongly against. [Female, 52 years, White, non-Hispanic, somewhat vaccine hesitant] (20)

Parents expressed numerous positive beliefs and attitudes that encouraged them to get the HPV vaccine for their adolescent children. They told stories about how family, friends, and personal experiences with HPV shaped how they thought about HPV infection and outcomes. Pediatrician recommendation was one of the strongest facilitators for influencing a parent’s decision-making.

#### Suggestions for Enhancing the HPV Vaccination Clinic Experience

Parents had ideas about enhancing communication with pediatricians and the clinic network that would help them feel more comfortable and informed about the HPV vaccine. Parents wanted HPV information and their pediatrician’s HPV vaccination recommendation for their child to be sent to them months before their child’s appointment:

*It would be much preferred if [the clinic] was able to send information to parents 4-6 months before each round of any vaccine is due…. facts (amazing how little any of us in this group can site facts for the HPV!) along with your personal pediatrician’s recommendation for your specific child: yay, nay, wait. Then it would be even better to have a chance to email or talk with the physician in advance of the appointment to ask questions. The HPV vaccine is a particularly awkward one to ask questions about in front of your 10 or 11 year old in the exam room.* [Female, 47 years, White, non-Hispanic, not vaccine hesitant] (21)

Information in advance of appt would be helpful because if I hadn’t already had experience with HPV vaccine I would have felt put on the spot when pedi asked if I wanted to give to him. [Female, 39 years, Black, non-Hispanic, not vaccine hesitant] (22)

It was also suggested that starting the conversation when the child is 9 years old would be helpful, along with the use of other clinic visits, such as the annual influenza vaccine appointment, to hand out informational materials. Parents wanted to be able to have a private dialog with the pediatrician if needed:

Provide info prior to the appointment, so parents can read and ask questions before going to the appointment. We don’t really want to talk about risks, consequences, etc. in front of the child. [Female, 45 years old, White, non-Hispanic, somewhat vaccine hesitant] (23)

Begin HPV discussion with patient family at 9 year well visit. Bring it up again at 10 year well visit. Make it clear that questions are welcome, as well as private dialog via phone, if that would help parent. There is never enough time in the exam room to discuss things. [Female, 47 years, White, non-Hispanic, not vaccine hesitant] (24)

There was a spectrum of content and time spent by pediatricians in discussing the HPV vaccine with parents. Some parents had ongoing conversations with the pediatrician, whereas others knew very little:

We were told on several visits about the seriousness of the virus and the potential to cause cancer. [Male, 45 years, White, non-Hispanic, not vaccine hesitant] (25)

I have very little information about the vaccine. The doctor briefly mentioned it and said it’s a good idea to do the series of three shots between the ages of 11-13. [Female, 40 years, White, non-Hispanic, somewhat vaccine hesitant] (26)

Parents reported a range of information they wanted from the pediatrician, everything from basic information to longitudinal research studies:

...a simple brochure with basic info and a guide to find other info would be nice to have [Female, 44 years, White, non-Hispanic, somewhat vaccine hesitant] (27)

I would like to see any studies that follow the patients that were the first recipients of the vaccine; I felt like the brochure that the pediatrician gave us to read was too dumbed down. I want real information, not a colorful brochure produced by the company that will profit from selling the vaccine. [Female, 52 years, White, non-Hispanic, somewhat vaccine hesitant] (28)

Parents suggested that partnering with school districts, having more information posted on the clinic network website, having an opt-in newsletter, and using social media to disseminate information would help them feel more informed about HPV. Parents were receptive to a clinic-sponsored HPV app if it included information they would want to receive from the pediatrician. Parents suggested including statistics on how widespread HPV is; how likely one is to be infected with it; what the risks of being infected with HPV are, especially for boys; the benefits of receiving the vaccine; data on side effects and adverse reactions; how many years it has been available; and how thoroughly tested the HPV vaccine is compared with other vaccines.

### Translating Findings Into DBCI Content and Feature Recommendations

After the focus group themes were identified, a wider research team, including pediatricians, behavioral scientists, statisticians, and designers, conducted multiple brainstorming sessions to translate the findings into DBCI content and feature recommendations focused on supporting the clinic network parents with their HPV vaccination decision-making processes. A total of 11 content and feature recommendations were suggested from qualitative themes (Table 2).

**Table 2:** Digital behavior change intervention (DBCI) content and feature design recommendations.

Recommendation	Description	Quote number^b^
Address HPV^a^ knowledge barriers	Address prominent HPV and HPV vaccine knowledge barriers (ie, child being too young or sexually inexperienced, boys not being eligible, safety and side effects, and effectiveness)	1, 2, 3, and 4
Use trusted sources to educate and correct misinformation	Use pediatricians to communicate information as they are trusted and respected sources for children’s health	17 and 18
Focus on HPV messaging that resonates with parents	Frame HPV information in a way that resonates with parents (ie, preventing cancer)	10, 11, 12, 13, and 14
Guide parents on navigating reputable HPV resources	Provide reputable HPV resources to parents and guide them in using best practices for navigating consumer health information on the internet	3, 4, 6, 7, 8, and 9
Describe reputable HPV research in a comprehensible format	Interpret and describe HPV scientific research in a comprehensible format (ie, plain language at sixth-grade level and infographics)	28
Communicate who is sponsoring the DBCI	Communicate that trusted sources (ie, pediatric clinic network) are sponsoring the product	5
Design for self-tailoring	Design for the spectrum of parent information needs from reviews of basic information to reviews of scientific studies	27 and 28
Design for a family audience	Design for engagement between family members, including adolescents who may influence their parent’s decision-making	19 and 20
Design for reflection	Give parents the opportunity to reflect on the health experiences of others in their personal and extended networks to increase salience and relevancy	15 and 16
Organize and prepare for the clinic visit	Prepare parents for their child’s clinic visit by having them organize their questions and concerns beforehand	21, 22, 23, and 24
Extend the clinic visit and enhance the clinic network	Create infrastructure that extends the clinic visit and leverages the clinic network so parents can better connect with others and needed information before and after the clinic visit	21, 22, 23, 24, 25, and 26

^a^HPV: human papillomavirus.

^b^The numbers refer to the numbered quotations in the paper.

#### Content Strategy Recommendations

The research team made six content strategy recommendations. First, the DBCI content should address the prominent HPV knowledge barriers expressed by the parents. These knowledge barriers stemmed from misinformation or a lack of information and included a child being too young or sexually inexperienced for the HPV vaccine, boys not needing or not being eligible for the HPV vaccine, the HPV vaccine having side effects that make it unsafe, and the HPV vaccine not being effective. The second content strategy recommendation was to increase the influence of the DBCI by having the network pediatricians be the voice that educates and communicates information, as they are highly trusted sources for obtaining quality advice about a child’s health. This might be accomplished through role modeling via video recordings or a frequently asked questions section that features photos and interviews with the pediatricians. The third content strategy recommendation was to frame information in a way that resonates with the parents, such as messaging, which focuses on cancer prevention and decreasing future risk. The fourth recommendation was to help parents navigate reputable HPV resources, as many parents felt it necessary to do their own HPV research before having their child vaccinated. This could involve educating on best practices for navigating consumer health information on the internet. The fifth recommendation was to help parents interact with HPV scientific research in a comprehensible format. A comprehensible format may involve featuring plain language interpretations and infographics of results from scientific studies. Finally, it was recommended to communicate who was sponsoring the app. The parents were skeptical of *big pharmaceutical companies* pushing anything related to the HPV vaccine, so making it clear that the app comes from trusted sources (ie, pediatric clinic network) may help improve its acceptability among parents.

#### Feature Design Recommendations

Five feature recommendations were made by the research team. The first was to design the DBCI for self-tailoring to meet the needs of individual parents. The HPV content parents wished to access differed in topic and depth as some parents were satisfied with the overview material, whereas others wanted to see scientific studies. Providing a self-tailored experience may be accomplished by presenting overview material but also offering links to reputable articles from the National Cancer Institute and CDC, giving parents the opportunity to explore a topic further if desired. The second recommendation was to include features that could engage a family audience, such as interactive games, as children’s attitudes toward the vaccine could influence their parents’ decision-making. The third recommendation was to have a feature where parents could reflect on the health experiences of others in their personal and extended networks. This might be advantageous for helping parents address anticipated regret and explore a more nuanced understanding of HPV. This might be accomplished by providing a guided prompt that parents can complete independently or with loved ones. As parents expressed limited time with the pediatricians during clinic visits, the fourth recommendation was to have a feature that organizes and records parent questions and pediatrician responses during the clinic visit. This may help increase the volume of information that can be discussed and reviewed. Finally, creating a DBCI that extends the clinic visit and enhances the clinic network infrastructure could address parents’ desire to have more interaction and communication beyond the clinic visit. For example, these features might include having personalized adolescent vaccination recommendations sent before a child’s appointment, having an opportunity to communicate about sensitive topics without the child being present, and connecting and learning from other parents in the clinic network.

### Feasibility of Skype Synchronous Text-Based Focus Groups

The synchronous text-based focus groups were effective in gathering insights from parents with adolescents belonging to a geographically diverse pediatric clinic network. Most parents were experienced using Skype *sometimes* (18/22, 82%). Parents requested a chat (text-only) format (8/17, 47%) over a call (audiovisual) format (1/17, 6%) for their focus group (Table 1). All parents successfully logged into their Skype accounts approximately 48 hours before their session and answered the welcome message as instructed. On the day of the session, all parents participated in their specified session. A total of 21 parents logged in and were ready to start on time (responded *yes* as instructed after reading the moderator’s introductory message). One parent was 4 minutes late to their session but was able to respond to the first topic posted in the chat. During one of the sessions, two accidental group calls were made a few minutes apart by a parent. However, the accidental calls did not disconnect any parents and did not cause any disruption beyond confusion for a few seconds. No calls were made to the technology facilitator. Cost savings were US $260 for each web-based session compared with the estimated costs for in-person sessions.

Key reflections on the feasibility and logistics of the format are highlighted in Table 3. Owing to the synchronous format, the sessions moved very quickly, with parents responding simultaneously at times. Preparing potential probes beforehand that could be easily modified and copy-pasted into the Skype chat was advantageous in keeping pace with participant responses. The format proved equitable with parents able to contribute to all relevant questions, and the Skype text bubbles, which occur when someone is typing, cued the study team into the cadence of posting questions. The format also produced automatic transcripts, allowing for immediate data analysis. As found in other studies, the anonymity provided by the format supported sensitive experiences being shared candidly [29,33]. For example, a parent discussed being sexually assaulted as an example of how rape might be a risk factor for acquiring HPV, and another discussed his own HPV cancer treatment. Web-based focus groups may be well-suited for discussions of HPV because of its association with sex, STIs, and reproductive cancers. An unexpected outcome of the web-based format was that parents shared articles and video clips in real time. It was beneficial for the study team to see examples of existing content that was influential in parents’ decision to vaccinate. The format increased the utility of parents to succinctly exemplify media, enabling more efficient understanding by the study team and easier translation to inform intervention content recommendations. At the conclusion of the focus groups, the study team sent an email to all participants clarifying HPV misinformation, answering any HPV questions they posed during the focus groups and directing them to reputable sources. The text-based format had several key weaknesses, including insights from facial expression, physical gesturing and prosody being difficult to gather and discern, and the format being labor intensive, with multiple team members needed to help moderate the high demands of processing simultaneous information.

**Table 3:** Skype feasibility and logistics.

Category and description			Findings and experience		Reflections			
					Strength			Weakness
**Format**								
	Participants were asked about their previous experience using Skype and were given the option to choose between a Skype chat–based focus group and a Skype audiovisual–based focus group.	Most participants had experience with using Skype before their session. Participants preferred a chat-based format over an audiovisual format. The original study plan to have half the sessions be audiovisual and half be text-only for comparative reasons was abandoned when scheduling for the large percentage of participants that requested a text-based format became prohibitive.		The automatic transcripts produced from the text-based format allowed for immediate qualitative data analysis supporting rapid formative research.			Insights from facial expression, physical gesturing, and prosody were difficult to discern and gather with the text-based format.	
**Attendance**								
	Participants were instructed to log into their Skype study accounts 48 hours before their session and answer the welcome message. Participants were instructed to log-in a few minutes before their specified session and reply to the moderator’s welcome instructions.	All participants successfully responded to the welcome message. On the day of the session, all participants successfully responded to the welcome instructions and attended their specified session. Most attended on time.		Participants were able to navigate the Skype chat function without issue. Attendance was high, possibly because of familiarity with the Skype platform and the ease of participating from a preferred location.			It was difficult to verify the identities of participants.	
**Confidentiality and anonymity**								
	Skype usernames (parents’ first name and ID number) and passwords were created for each participant and deleted at the conclusion of each session.	By only using the participants’ first names, they were able to recognize when someone was addressing them but still keep their identity anonymous.		Sensitive experiences were shared candidly.			It was difficult to verify the identities of participants.	
**Moderator considerations and cadence**								
	A total of 4 team members ran each session: the lead moderator, a tech facilitator, and 2 submoderators.	At times, the session moved very quickly, with participants answering questions simultaneously. Skype chat provided text bubbles when a participant was typing which aided the research team in establishing the cadence of asking questions. Preparing potential probes that could be easily modified and copy-pasted into the Skype chat proved advantageous for keeping pace with participants. Having participants use Skype on a laptop or desktop with a connected keyboard rather than on their phone proved advantageous for more uniform and rapid response time.		All participants were able to contribute to all relevant questions at their own speed.			The type-based format took more team members to moderate than an in-person session because of the high demands of processing incoming information spurred by simultaneous typing and posting.	
**Group dynamics**								
	Unexpected outcomes occurred from the synchronous web-based format.	Participants shared HPV^a^ articles and video clips that influenced their decision to vaccinate in real time. At the conclusion of the focus groups, the team sent an email to all participants clarifying HPV misinformation, answering any HPV questions they posed during the focus groups and directing them to reputable sources.		It was beneficial for the research team to see examples of actual content that influenced participants’ decision to vaccinate. The format increased the utility of participants to succinctly exemplify media, enabling more efficient understanding by the study team.		It was important to clarify misinformation after the sessions as participants shared articles that used persuasive tactics of expert opinion and pseudoscience to discredit the HPV vaccine.		
**Tech support and disruption**								
	A ‘how to download and use Skype’ guide with screenshots was distributed before the sessions. A technology facilitator was available during the sessions for support.	No assistance was needed downloading or operating Skype. Two brief accidental calls occurred during one session but did not cause major disruption or require intervention. Technology support was not actively needed during the sessions.		Participant familiarity and experience with Skype supported efficient operations.		Although not needed, it may have proved difficult to help participants navigate technical challenges remotely.		
**Cost savings**								
	The costs of conducting the focus groups web-based versus in person were compared.	Cost savings were estimated at US $260 for each session, accounting for transcription, parking reimbursements, and clinic staff after-hours pay.		Skype is a free platform and provides cost savings compared with in-person methods.		The format may bias participation toward those with access to a computer and internet and who feel comfortable with web-based communication.		

^a^HPV: human papillomavirus.

## Discussion

### Principal Findings

This study highlights the factors that influence HPV vaccine decision-making among a group of mostly White, non-Hispanic, and educated parents whose adolescents were patients at a large urban pediatric clinic network in the United States. The findings from these synchronous text-based focus groups align with findings found in other HPV vaccination studies conducted with parents [15,18,28,47,48]. Even among these highly educated individuals, where many expressed interactions with and access to health care professionals, there was confusion, misinformation, and a lack of knowledge regarding HPV and the HPV vaccine. This has been a consistent challenge since the introduction of the first HPV vaccine in 2006 [49,50]. Similar to other studies, parents generally had favorable attitudes toward adolescent vaccinations but differed in how they viewed the HPV vaccine [47]. Also consistent with prior findings is the role pediatrician recommendation serves in positively influencing HPV vaccination parental decision-making [15,48]. Personal or familial experience with HPV had a significant influence on HPV vaccination attitudes and beliefs, even leading some parents to become outspoken advocates for the vaccine. Conducting their own research on the web was an important step in parent HPV vaccination decision-making and could lead to missed opportunities for vaccination if the parent had not yet researched HPV or was not prepared to discuss their outstanding questions with the pediatrician at the clinic visit. It is not surprising that the parents had a difficult time discerning reputable web-based research, as antivaccination websites often use expert opinion (using the title of *Dr*) and pseudoscientific evidence (confusing correlation for causation) as persuasive tactics [51].

These results indicate that parents want more communication from their child’s pediatrician and have certain questions and advice they are actively seeking. Pediatric clinic networks have the opportunity to cultivate credible, relatable, and understandable HPV information for distribution to parents and patients before a clinic visit to increase HPV vaccination rates. Content and feature recommendations from this study can provide guidance for researchers and developers involved in the creation of HPV-focused apps for parents. These recommendations are largely consistent with general recommendations for digital health interventions and are related primarily to mitigating concerns and misinformation, providing authentic and persuasive messages, providing user control in inquiries of content breadth and depth, and facilitating a move to immediate action (vaccination appointments) [52,53]. In addition, features and content that incorporate personalization, reinforcement learning, social support, credibility of sources, and focus on simple and consistent interface esthetics, easy-to-use navigation, and multimedia messages have been found to influence and improve user participation across health topics [54]. The recommendations emerging from this work provide insight but should not be regarded as definitive. Future research and development that uses well-validated theory and empirically-based development frameworks to design and formatively evaluate proofs-of-concept and prototypes is recommended [22-25]. In this regard, this study represents an important initial needs assessment step to give voice to the patient and parent experience and translate it into a responsive intervention.

Recent events of the COVID-19 pandemic have seen a dramatic movement toward web-based communication across transaction domains, including research protocols. Focus groups are well accepted as a needs assessment method, and now platforms such as Zoom and WebEx are ubiquitous and offer multiple features, including video, chat, questions and answers, and break-out room features to facilitate them. This study presents results that can provide useful insights when considering the efficacy of synchronous text-based focus groups and the advantages of this method. The preference of the participants to opt for text-based communication was unexpected; however, it was consistent with a desire for confidentiality, anonymity, convenience, and avoidance of video-related bandwidth and other technical problems. Furthermore, the text-based format provided response equity, enabling each participant to submit responses irrespective of their comfort in group situations. Parents participating in these focus groups approximated the demographics of the clinic network population as predominately White, non-Hispanic, and privately insured. However, a limitation of the study is that the web-based format may have excluded participation from parents with lower socioeconomic status or those who did not feel comfortable with web-based communication. The results may not be generalizable to parents who are younger, have lower educational attainment, who are publicly insured, from rural communities, or are not White and non-Hispanic. Future research addressing these populations is recommended. Although seemingly amenable to information gathering, this study was exploratory and did not determine personal determinants that might aid recruitment into such groups or potentially bias information gained in this type of forum. Further concerns about this approach are the impact of lack of nonverbal feedback from the group and the potential challenge of ensuring accountability of participants in focusing on the discussion. Further research in this regard is recommended. The method did offer significant parsimony from a logistic perspective, as the text-based approach had distinct advantages for streamlining data processing, management, and analysis when compared with audio or video recordings.

### Conclusions

Synchronous text-based focus groups conducted via Skype are feasible for conducting DBCI formative research on HPV vaccination decision-making. Among this well-educated and well-resourced parent sample, there were barriers such as misinformation and facilitators such as pediatrician recommendations that influenced HPV vaccination decision-making. Parents want easy-to-understand and relevant HPV vaccination advice from their child’s pediatrician and an enhanced clinic visit experience, which lets them access and connect to tailored information before and after clinic visits. The results gathered provide guidance for content and features that may inform a more responsive DBCI to address HPV vaccination decision-making among parents.

## Abbreviations

CDC: US Centers for Disease Control and Prevention
DBCI: digital behavior change intervention
HPV: human papillomavirus
STI: sexually transmitted infection
Tdap: tetanus, diphtheria, and pertussis
